# Case Report: Scalp pityriasis versicolor may be a neglected problem

**DOI:** 10.3389/fped.2024.1361225

**Published:** 2024-06-19

**Authors:** Ya Bin Zhou, Jin Jing Chao, Lin Ma, Yuan Yuan Xiao

**Affiliations:** Department of Dermatology, National Key Discipline of Pediatrics, Key Laboratory of Major Diseases in Children, Ministry of Education, National Center for Children’s Health, Beijing Children’s Hospital, Capital Medical University, Beijing, China

**Keywords:** pityriasis versicolor, tinea versicolor, scalp, *Malassezia*, children

## Abstract

Pityriasis versicolor, a common skin fungal infection, is typically observed on trunk and limb skin. Here, we highlight an unusual presentation: scalp involvement, often overlooked due to its asymptomatic, mildly scaly patches. We report four pediatric cases, emphasizing the potential underestimation of this scalp variant. This case series underscores the importance of considering this diagnosis in patients with unexplained scalp hypopigmentation, especially in males with short hair who may readily notice these subtle changes. The report contributes to the understanding of this variant's clinical presentation and emphasizes the need for awareness among clinicians to ensure accurate diagnosis and appropriate management.

## Introduction

Pityriasis versicolor, also known as tinea versicolor, is a prevalent superficial cutaneous fungal infection caused by dimorphic lipophilic and lipid-dependent yeasts in the genus *Malassezia* (formerly known as *Pityrosporum*), notably *Malassezia globosa*, *Malassezia furfur* and *Malassezia sympodialis* (1). This condition occurs globally, with significantly high prevalence rates observed in hot and humid climates, reaching up to 50% in some tropical countries (2). In contrast, countries like Sweden report much lower prevalence rates, as low as 0.5% (3). Pityriasis versicolor primarily affects adolescents and young adults, possibly due to increased sebum production within these age groups (4). It is less common in children, but the causative species appear to be the same in children and adults (5). Clinically, it is characterized by mildly scaly hypopigmented or hyperpigmented macules/patches, typically found on areas of the skin rich in sebum production such as the trunk (especially the upper part), neck, shoulders, and upper arms (1). However, it can also occur in unusual regions of the body, including the face, scalp, arms, legs, intertriginous sites, genitalia, areolae, and palms and soles (6). In this case series, we present instances of scalp pityriasis versicolor in pediatric patients, highlighting a rare distribution of this common disease.

## Case description

This case series explores four distinct cases of pediatric pityriasis versicolor, ranging from infants to teenagers. All patients exhibited gradually emerging, asymptomatic hypopigmented lesions on the scalp. Notably, the lesions in the older children were coincidentally identified during routine haircuts. Significantly, none of these patients reported any history of animal contact and maintained overall good health.

**Figure 1:**
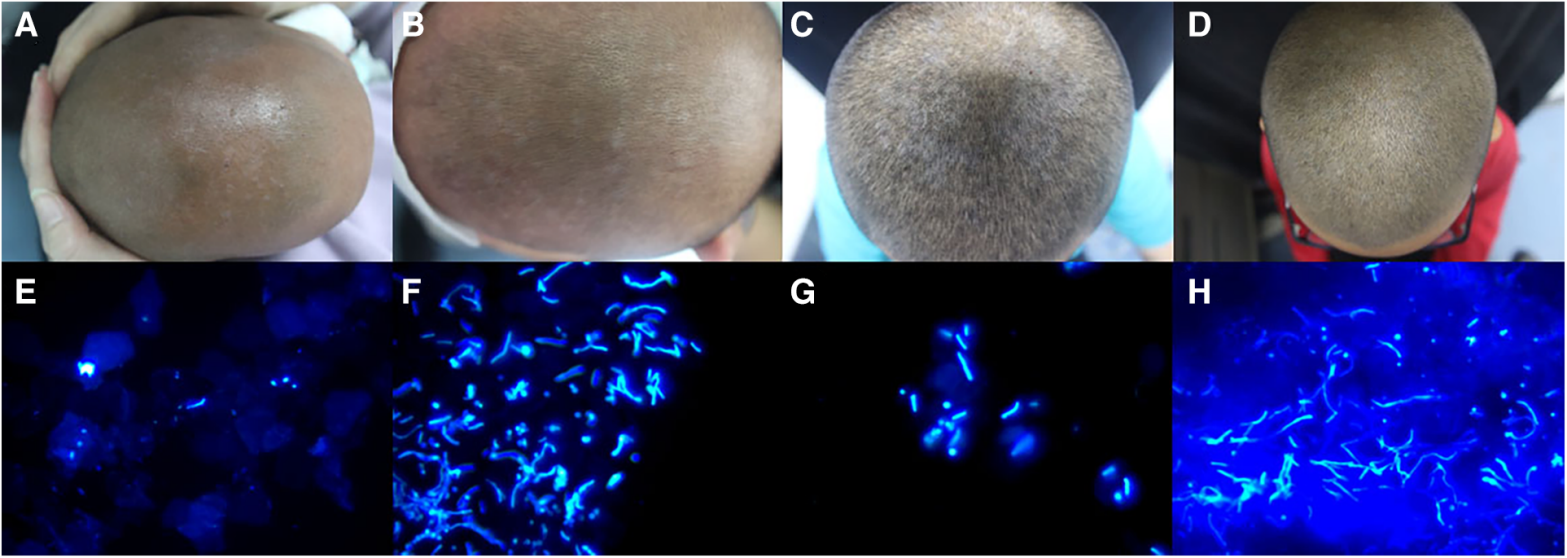
Clinical manifestations of patient 1 (**A**), patient 2 (**B**), patient 3 (**C**), and patient 4 (**D**) direct microscopic examination of lesions from patient 1 (**E**), patient 2 (**F**), patient 3 (**G**), and patient 4 (**H**) (calcofluor white staining, original magnification ×400).

Upon examination, all patients exhibited distinctive, well-defined, round-to-oval, hypopigmented macules and patches on the scalp (Figures 1A–D). No involvement of other body sites was observed in any of the patients. Direct microscopic examination of these lesions revealed the presence of characteristic thick-walled yeasts and short angular hyphae, reminiscent of the classic “spaghetti and meatballs” appearance (Figures 1E–H). Based on these findings, all patients were diagnosed with pityriasis versicolor.

Treatment strategies are outlined in Table 1. Patient 1 and 2 were treated with topical terbinafine hydrochloride cream twice daily for 6 and 8 weeks respectively, while Patient 3 and 4 received topical ketoconazole shampoo twice weekly for 8 and 12 weeks respectively. Remarkably, no adverse reactions were observed during the course of topical antifungal therapy for any patient. At the 3-month follow-up, no recurrence was reported, indicating the efficacy and safety of the prescribed topical antifungal treatments for pediatric pityriasis versicolor.

**Table 1:** Characteristics of patients with scalp pityriasis versicolor.

Patient no.	Age	Sex	Treatment	Time of therapy (weeks)	Adverse reaction	Follow up
1	8 m	Male	Terbinafine cream	6	None	No recurrence
2	7 m	Male	Terbinafine cream	8	None	No recurrence
3	14 y	Male	Ketoconazole shampoo	8	None	No recurrence
4	8 y	Male	Ketoconazole shampoo	12	None	No recurrence

m, month; y, year.

## Discussion

*Malassezia* species, unique fungi residing on the skin of humans and non-human mammals, are notably absent from environmental sources. With the exception of *Malassezia pachydermatitis*, these fungi specifically inhabit sebum-rich areas of the skin, relying on various fatty acids for growth. Consequently, culturing *Malassezia* species requires the addition of specific compounds like Tween, oleic acid, and bile salts to the medium (4, 7).

Within the *Malassezia* genus, 18 species are identified, ten of which are associated with human skin, while the remaining eight inhabit non-human animal skin (8). The most recent human-derived discovery was *Malassezia yamatoensis* in 2004, isolated from a patient with seborrheic dermatitis (9). Although typically commensal, *Malassezia* species can induce conditions like pityriasis versicolor, seborrheic dermatitis, *Malassezia* folliculitis, and atopic dermatitis (4). *M. restricta* and *M. globosa* dominate healthy and diseased skin, but their ratios vary: *M. restricta* surpasses *M. globosa* in seborrheic dermatitis, whereas *M. globosa* dominates in pityriasis versicolor (10).

Pityriasis versicolor shows a slightly higher prevalence in men, likely attributed to increased sebaceous activity among males (1). Interestingly, there are limited documented cases of scalp pityriasis versicolor, predominantly involving male patients, as observed in our report (11–15). This prevalence among males could be linked to the common practice of shorter haircuts in men, increasing the likelihood of detecting scalp pityriasis versicolor. Notably, our older patients noticed the condition during haircuts, underscoring the importance of this factor.

The location of pityriasis versicolor lesions varies based on the individual's age, with the face and neck being more prevalent sites in children (11). While scalp involvement is rare in both children and adults, seborrheic dermatitis, another *Malassezia*-associated skin condition, commonly affects the scalp (16). A likely explanation for this underreporting lies in the distinctive characteristics of the two conditions. Seborrheic dermatitis typically manifests as itching and scaling, accompanied by well-defined erythematous patches. These symptoms readily draw patients' attention. In contrast, scalp pityriasis versicolor presents as mildly scaly hypopigmented or hyperpigmented macules/patches, often escaping notice. Consequently, the inconspicuous nature of this condition might contribute to its underdiagnosis.

Pityriasis versicolor lesions display a spectrum of colors, ranging from nearly white to reddish-brown or fawn. Notably, hypopigmentation, especially discernible on dark skin, has been identified as an independent variant (17). While children are less commonly affected by this condition than adults, it is intriguing that 72% of pediatric cases present in the hypopigmented form (18), a consistency found in our study where all pediatric scalp pityriasis versicolor cases demonstrated this hypopigmented variant. The precise mechanism behind this hypopigmentation remains elusive; however, recent literature posits a hypothesis. It suggests that *Malassezia* spp. could produce malassezin, a substance capable of stimulating melanocyte apoptosis, and azelaic acid, which has the potential to inhibit tyrosinase activity, thus contributing to the observed hypopigmentation (19).

Diagnosing pityriasis versicolor typically relies on clinical observations, rooted in the condition's distinct features. However, the diverse manifestations of tinea versicolor can perplex inexperienced clinicians. Using a Wood lamp, lesions may exhibit fluorescence in shades of gold-yellow, yellowish-green, or coppery-orange, although not all lesions fluoresce (1). Microscopic examination reveals short, stubby hyphae mingled with spore clusters, creating the characteristic “spaghetti and meatballs” appearance (20). Culturing or molecular analysis of *Malassezia* species from skin scrapings lacks diagnostic value and isn't part of pityriasis versicolor's diagnostic protocol (20). Although dermoscopy aids in confirming scaling, it doesn't pinpoint specific diagnostic markers (21).

When dealing with pityriasis versicolor, initial treatment typically involves topical antifungals, with systemic antifungals reserved for severe or persistent cases. Effective topical treatments include creams, lotions, and shampoos, applied once or twice daily for varying durations, often swiftly alleviating clinical symptoms. Non-specific treatments, such as selenium sulphide (available in lotions, creams, or shampoos), zinc pyrithione, propylene glycol, and Whitfield's ointment, have demonstrated efficacy against the condition (22, 23). Recent studies have primarily focused on ketoconazole and terbinafine among topical antifungals (23). However, patient adherence might be influenced by the need for frequent applications or mild skin irritation. The efficacy of oral agents like ketoconazole, itraconazole, and fluconazole is well-established (1). Notably, oral terbinafine, effective for various superficial fungal infections, does not effectively treat pityriasis versicolor (24). This limitation arises because oral terbinafine is not excreted through sweat, preventing the attainment of fungicidal levels in the stratum corneum (24). In our case series, older children opted for topical ketoconazole shampoo, mainly due to the discomfort associated with applying cream on the scalp.

In conclusion, we have documented four cases of pityriasis versicolor on the scalp, representing an uncommon manifestation of this common diseases. Due to the scalp's concealment by hair and the asymptomatic nature of pityriasis versicolor, characterized by hypopigmented or hyperpigmented macules/patches, it often goes unnoticed by patients. We posit that scalp pityriasis versicolor might be significantly underestimated.

## Data Availability

The original contributions presented in the study are included in the article/Supplementary Material, further inquiries can be directed to the corresponding author.
